# Effectiveness of scalpel debridement for painful plantar calluses in older people: a randomised trial

**DOI:** 10.1186/1757-1146-4-S1-O23

**Published:** 2011-05-20

**Authors:** Karl B Landorf, Adam Morrow, Martin J Spink, Chelsey L Nash, Anna Novak, Adam R Bird, Julia Potter, Hylton B Menz

**Affiliations:** 1 Department of Podiatry, Faculty of Health Sciences, La Trobe University, Victoria 3086, Australia; 2 Musculoskeletal Research Centre, Faculty of Health Sciences, La Trobe University, Bundoora, Victoria 3086, Australia; 3 School of Health Sciences, University of Southampton, Southampton, SO17 1BJ, UK

## Background

Plantar calluses are often associated with foot pain, which can have a detrimental impact on the mobility and independence of an older person. Scalpel debridement is a key management strategy for painful corns and calluses, however the effectiveness of this treatment in older people has not been rigorously investigated. Therefore, we conducted a parallel-group randomised trial to evaluate the effectiveness of scalpel debridement in reducing pain associated with forefoot plantar calluses.

## Methods

Eighty participants aged 65 years and older with painful forefoot plantar calluses were recruited from a university podiatry clinic, a retirement village, and community advertisements between May 2006 and November 2008. Participants were randomly allocated to one of two groups: (i) normal (experimental) scalpel debridement or (ii) sham (control) scalpel debridement. Participants were followed for six weeks after their initial intervention appointment. Both participants and assessors were blinded to the intervention. The primary outcomes measured were the difference between groups in pain (measured on a 100 mm visual analogue scale) and barefoot peak plantar pressure (measured using a MatScan® System). Secondary outcome measures included tests of balance and functional ability. The sample size was pre-specified using an appropriate sample size/statistical power calculation. Statistical comparison between the groups was made using a linear regression approach to ANCOVA and analysis was by intention to treat. The trial was registered with the Australian Clinical Trials Registry, ACTRN12606000176561.

## Results

Both groups experienced large decreases in pain following intervention (up to a 41.9 mm decrease in pain on a VAS). A systematic, but small beneficial effect on pain was noted in favour of the normal scalpel debridement group immediately post-debridement to 4 weeks post debridement (from 6.0 to 7.2 mm ANCOVA adjusted mean difference between groups). These values, however, are likely to be below a minimal important difference (i.e. clinically important to a patient) and there were no statistically significant differences (p<0.05) in pain levels between the two groups at any of the primary endpoints (immediately post-debridement, and at 1, 3 and 6 weeks post-debridement). In addition, there were no differences in peak plantar pressure or balance and functional ability between the two groups at any time-points. There were no adverse events of note.

## Conclusions

The findings of this trial indicate that scalpel debridement of painful plantar calluses has minimal effect on its own. While we found a systematic effect favouring scalpel debridement, the benefits were small and not statistically significant. It is likely that scalpel debridement offers minimal pain relief, so other aspects of conservative care of painful calluses (e.g. padding) in addition to debridement may provide greater benefits that are clinically worthwhile to patients. In the absence any safety issues, we conclude that scalpel debridement of painful plantar calluses is of small benefit to patients, but other forms of conservative care in combination with scalpel debridement may provide clinically worthwhile benefits to patients.

